# Status Quo of Professional–Patient Relations in the Internet Era: Bibliometric and Co-Word Analyses

**DOI:** 10.3390/ijerph16071183

**Published:** 2019-04-02

**Authors:** Zekun Wang, Zhaohua Deng, Xiang Wu

**Affiliations:** School of Medicine and Health Management, Huazhong University of Science and Technology, Wuhan 430030, China; u201612699@hust.edu.cn (Z.W.); zh-deng@hust.edu.cn (Z.D.)

**Keywords:** Professional–Patient Relations, Internet, Bibliometrics, Cluster analysis, Social networking

## Abstract

*Background*: Incidents of violence against medical staff have increased in intensity, showing the deteriorating relationship between doctors and patients in China over the past few years. In addition, professional–patient relations have been significantly affected in the Internet era in China, which has attracted great attention from many scholars. This study aims to analyze the research status of professional–patient relations in the Internet era in China and further reveal its research pattern and trends. *Methods*: This study collected journal articles published during the past 21 years from the Wanfang Data Knowledge Service Platform. Then, bibliometric analysis was carried out, including publication growth, core author and collaborative degree, highly cited papers, journal distribution, and institution distribution analyses. We also analyzed the subject heading–source literature matrix and co-occurrence matrix of keywords through hierarchical cluster, social network, and strategic diagram analyses. *Results*: The number of articles has continually risen since 1998, which follows the growth law of literature. Furthermore, the distribution of these studies obeys Bradford’s law of scattering, and mainly concentrates on the fields of medicine and health technology. The distribution of high-frequency keywords follows Zipf’s law. *Conclusions*: We identified eight focal research directions, namely: website building (especially for professional–patient interaction), telemedicine, professional–patient communication and network public opinion, professional–patient contradiction and health education, new media, follow-up interaction platform, healthcare reform and computer network, and medical ethics.

## 1. Introduction

We have witnessed rapid worldwide development of the Internet. The number of Internet users in North America reached 346 million by June 30, 2018, with the Internet penetration rate being 95% [1]. As for China, the Internet has experienced a breakneck development over past decades, with the number of Chinese netizens reaching 802 million in June 2018. Furthermore, the Internet penetration rate, which was 57.7%, exceeded Asian and global average levels and is expected to grow steadily. At the same time, the number of mobile netizens reached 788 million, which made the rates of mobile phone usage reach a new record [2]. According to the “Internet Plus” policy proposed in the government work report, the medical industry is facing great opportunities [3]. More doctors and patients are benefiting from various Internet services, such as professional–patient interaction, knowledge-based, and comprehensive medical services.

As an essential factor in the medical environment and China’s healthcare reform [4], professional–patient relations are inevitably affected by the Internet [5]. Incidents of violence against medical staff have increased in intensity, showing deteriorating professional–patient relations under the traditional medical mode in China over the past few years [6,7,8,9,10]. However, the Internet undoubtedly brings important opportunities for the improvement of professional–patient relations, such as professional–patient interaction websites, new media, and the implementation of a hospital information system [4]. In the Internet era, patients have an empowered voice, have begun to make shared medical decisions with doctors, and will replace doctors as the center of the medical service system in the 21st century [11]. As a result, the development of the Internet seemingly breaks the traditional professional–patient pattern [12].

In addition, professional–patient relations in the Internet era have attracted significant attention from many scholars and become a research hotspot. Subsequently, a sharp rise in the number of articles on this topic has occurred, and some of these articles are quite significant and influential (e.g., Lai and Yang [13]; Dai [14]; Wang [15]). These articles explore how to effectively improve professional–patient relations in the Internet era in line with the current health situation in China. For example, under the implementation of a hierarchical medical system, the use of Internet technology to improve professional–patient relations is discussed [16]. New healthcare reform priorities include increasing patient satisfaction with healthcare and improving the quality and safety of care [4]. China is in the midst of reforming and modernizing its health care system. At the same time, China is also the largest developing country in the world, facing the dilemma of using extremely limited and unevenly distributed health resources to solve the medical problems of one-fifth of the world’s population. Its exploration of how to improve professional–patient relations will help other developing countries facing similar dilemmas to improve their professional–patient relations. In addition, such an exploration provides references for scholars from all over the world to study the professional–patient relations model in developing countries.

However, until now, only a brief overview of professional–patient relations in the Internet era in China has been available, and a review of previous related literature on the topic shows some research limitations. That is, few Chinese scholars have explored the research structure based on quantitative methods. The primary goal of the present study is to analyze the research status of professional–patient relations in the Internet era in China and further reveal its research pattern and trends, as well as address the limitations by conducting a comprehensive analysis of the professional–patient relations in the Internet era in China based on bibliometric and co-word analyses.

## 2. Materials and Methods

### 2.1. Search Strategy

**Figure 1:**
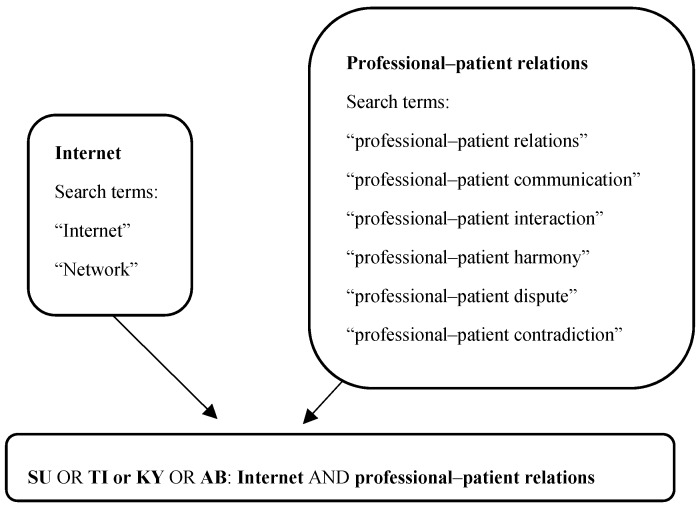
Search strategy used for searching for articles. *Note*: SU: subject; TI or KY: title or keywords; AB: abstract.

We performed a systematic literature review following the Preferred Reporting Items for Systematic Reviews and Meta-Analyses (PRISMA) guidelines [17]. Here, we focused on searching for articles related to professional–patient relations in the Internet era in China. The search strategy is as follows, and is provided in Figure 1.

(SU: ((Internet or network) and (professional–patient relations or professional–patient communication or professional–patient interaction or professional–patient harmony or professional–patient dispute)) + (TI (title) or KY (keywords)): ((Internet or network) and (professional–patient relations or professional–patient communication or professional–patient interaction or professional–patient harmony or professional–patient dispute)) + AB (abstract): ((Internet or network) and (professional–patient relations or professional–patient communication or professional–patient interaction or professional–patient harmony or professional–patient dispute)))*Date: –2018

Given that the review aimed to provide a quality overview of professional–patient relations in the Internet era in China, we included only journal articles written in Chinese with a more academic nature than other journal articles. The term “Internet” was combined with the term “professional–patient relationship” to limit the scope to the professional–patient relations field.

The Wanfang Data Knowledge Services Platform is one of the most comprehensive databases for medical journals in China. Since 2007, it has exclusively included a series of journals of the Chinese Medical Association. Furthermore, we retrieved journal articles with the advanced search function in the China Academic Journals (CNKI) and Wanfang Data Knowledge Services Platform. We chose the Wanfang Data Knowledge Services Platform as the data source because the amount of literature embodied in it is more than that in CNKI.

### 2.2. Data Collection

**Figure 2:**
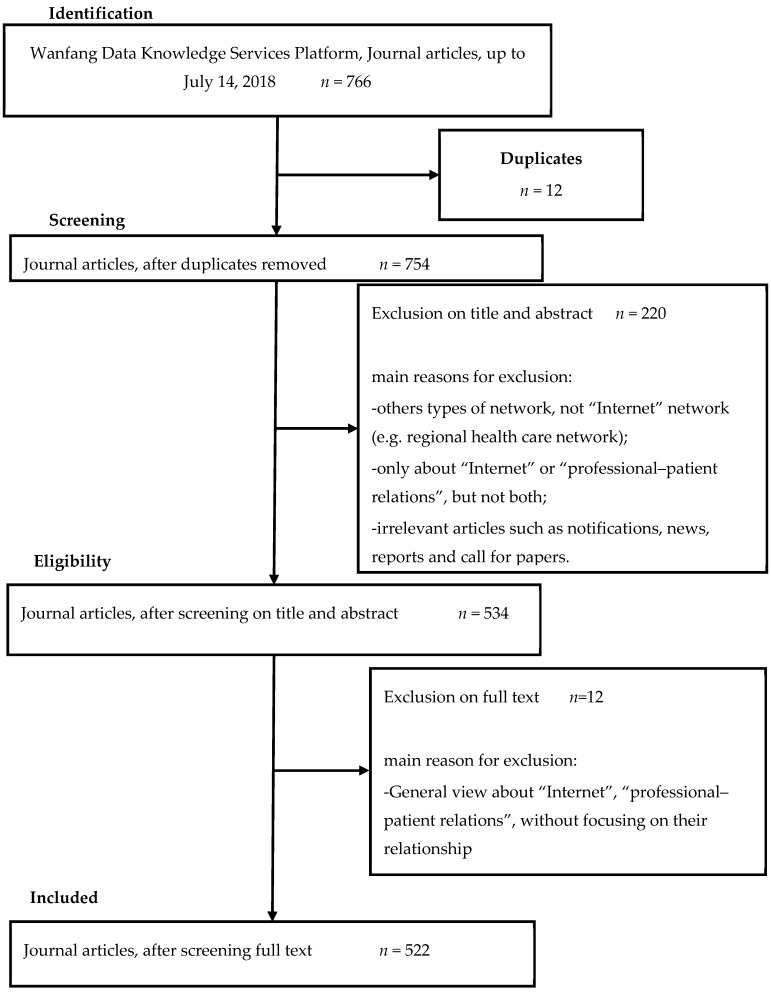
Search strategy (Preferred Reporting Items for Systematic Reviews and Meta-Analyses), with reasons for exclusion and inclusion of articles added.

We screened the titles and abstracts of the identified articles to assess inclusion in the full review (Figure 2). Articles were included in the analysis if they involved at least one of the following:

(1) A discussion on how to promote the harmonious development of professional–patient relations in the Internet era;
(2) A general review of how the Internet affects professional–patient relations;
(3) A detailed exploration of how the Internet affects professional–patient relations in a specific dimension, such as technology, media, and information resources;
(4) The use or development of Internet products to improve professional–patient relations, such as applications, a follow-up interaction platform in the hospital, and an online health community.

Then, we extracted and downloaded the bibliographic records of 522 articles selected for subsequent bibliometric and co-word analyses, to analyze the research status, hotspots, and trends of professional–patient relations in the Internet era in China.

### 2.3. Method of Data Analysis

We carried out the statistical analysis using BICOMB2 (China Medical University, Shenyang, China), Microsoft Excel 2016 (Microsoft Corporation, Washington, DC, USA), and IBM SPSS Statistics 24 (IBM Corporation, New York, NY, USA). We also conducted five stages for the hierarchical cluster and strategy diagram analyses.

First, we sorted out the keywords and calculated the frequencies of each keyword with BICOMB2. We initially imported the bibliographic records of these articles into BICOMB2. To get more precise results, we subsequently standardized some keywords by merging the synonyms (e.g., “Internet + medical” and “Internet medical” were replaced by “Internet + medical,” “communication” and “professional–patient communication” were replaced by “professional–patient communication”) and excluded the general terms that were meaningless or too broad (e.g., study, analysis, influence, and apply). Finally, after sorting out the keywords, we calculated the frequencies of all keywords.

Second, we chose 22 high-frequency keywords from the total keywords using the g-index [18]. Then, we analyzed the distribution of high-frequency keywords and chose 22 keywords with frequencies of no less than eight to generate a 22 × 22 co-occurrence matrix for the social network analysis, and a subject heading–source literature matrix for the hierarchical cluster analysis [19].

Third, we conducted a hierarchical cluster analysis using SPSS 24, with average linkage (between groups) as the cluster method and squared Euclidean distance as the distance measure [20]. Thus, keywords with higher correlations with one another are more likely to be put into the same cluster than those with lower correlations. Different cutoff steps may be set up in the hierarchical clustering to get different clustering results, which can provide more explanations of the correlation between keywords or themes [21]. Each cluster denotes a possible research theme.

Fourth, we calculated the centrality and density of each cluster using Excel 2016. Density corresponds to the internal correlations of the cluster, whereas centrality corresponds to the weight of the external links of the cluster [22]. The theory of strategic coordinates, put forward by Law et al. in 1988 [23], describes the correlation between contents in a particular field and the mutual influence between different fields. We used the following formulas to calculate density and centrality [24]:

(1) $$\mathrm{Density}=\frac{\sum_{i,j\in \varphi_{s}}E_{ij}}{n-1}\left(i\neq j\right),$$

(2) $$\mathrm{Centrality}=\frac{\sum_{i\in \varphi_{s},j\in \left(\varphi -\varphi_{s}\right)}E_{ij}}{N-n};\;E_{ij}=\frac{{\left(C_{ij}\right)}^{2}}{C_{i}*C_{i}},$$

where $C_{ij}$ stands for the co-occurrence frequency of keywords i and j, and $C_{i}$ stands for the frequency of keyword i. $E_{ij}$ belongs to [0,1]. In addition, φ represents the entire keyword network, whereas $\varphi_{s}$ is a certain cluster. N is the number of total keywords in the entire network, and n is the number of keyword(s) in a certain cluster.

Finally, we drew a strategic diagram to intuitively present the hotspots and trends of professional–patient relations research in the Internet era in China [22]. The strategic diagram uses a two-dimensional space to plot clusters according to their centrality and density. Therefore, the theme clusters located in four quadrants, with different centralities and densities, can indicate the developing status of research themes.

## 3. Results

### 3.1. Literature Distribution

#### 3.1.1. Publication Growth Analysis

**Figure 3:**
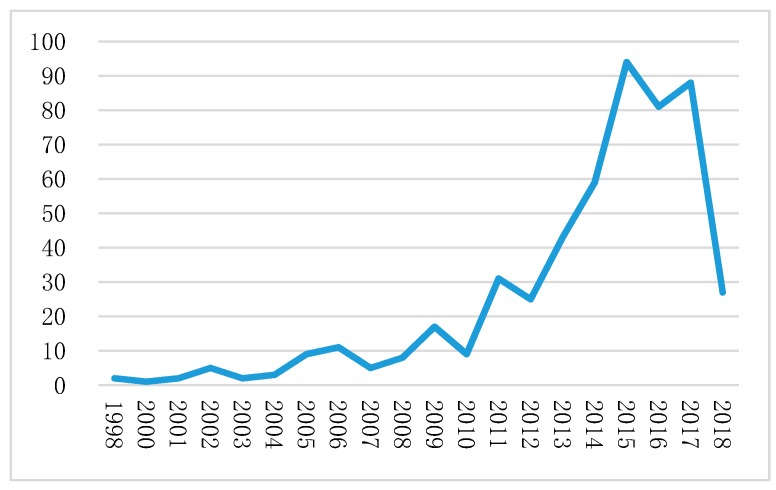
Number of publications of each year (1998–14/7/2018).

According to the retrieved results, the earliest article on the topic, embodied in the Wanfang Platform, appeared in 1998. Figure 3 shows the publication output from 1998 to 2018. The number of articles has risen yearly and increased from 1 in 1998 to 88 in 2017. Moreover, the number significantly increased from 5 in 2007, and reached a peak of 94 in 2015.

**Figure 4:**
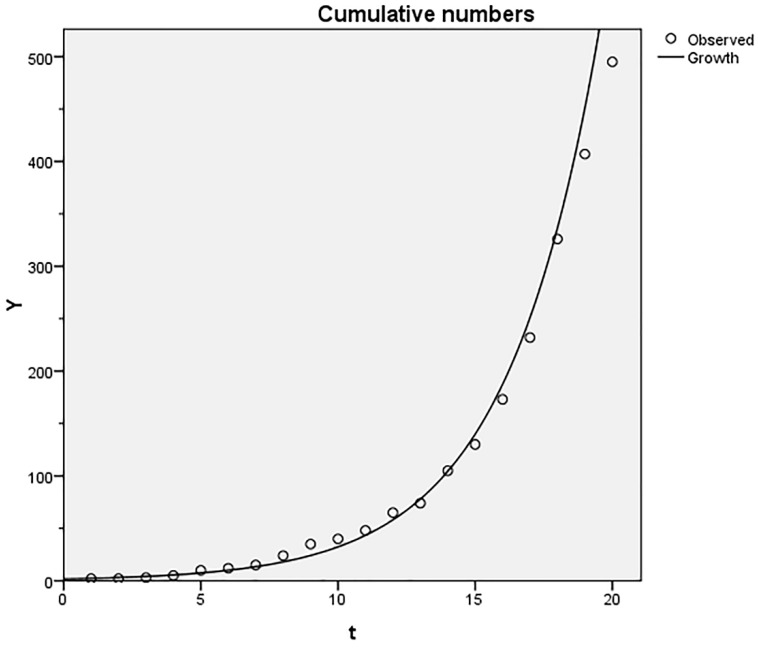
The relationship between the cumulative number of publications and years, 1998–2017.

Figure 4 shows that the cumulative number of publications continually grew from 1 to 495 in 2017. We obtained a literature growth curve by directly fitting the following equation:

Y = 2122.611/(1 + 1218.277e − 0.297t), *R*^2^ > 0.998, (3)

where Y is the cumulative number of articles and t (time) is the number of years since 1998 (shown in Figure 4). Moreover, combined with equation:

*t* = ln(1218.277)/0.297 = 23.923 > 21, (4)

we can infer that the publication output on the topic is still in a period of rapid growth since 1998 [25,26].

#### 3.1.2. Core Author and Collaborative Degree Analyses

There are 507 researchers listed as the first author, accounting for 40.37% of the total 1256 authors. In addition, 1195 authors, comprising 95.14% of the total authors, published only one article. Some authors published four articles at most. We calculated the minimum output of the core author with the equation:

(5) $$M\approx 0.749\sqrt{N},$$

where M is the minimum output of the core author and N is the maximum output of the core author [27]. According to the law of price, authors with articles more than M should be considered core authors. Therefore, the result is 1.498, which means that the publication output of every core author is not less than two. In this study, 61 core authors have published 132 articles, accounting for 25.29% of the total articles. This percentage is far less than 50%. According to the law of price, this result shows that the core authors group has not been formed, and the publication output of the core authors should be increased [24].

**Figure 5:**
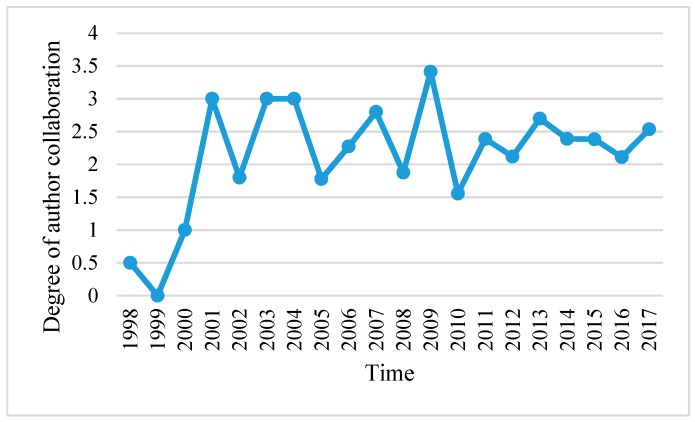
The degree of author collaboration by year (1998–2017).

Overall, 1256 authors have published 522 articles, indicating that the average degree of author collaboration was 2.406. This result shows that Chinese scholars do not collaborate intensively compared to international publications. Figure 5 shows that the degree of author collaboration was broadly on the rise from 1998 to 2017. Notably, the average collaborative degree of the author significantly fluctuated from 1999 to 2010.

#### 3.1.3. Highly Cited Paper Analysis

Given that the citation frequency of highly cited papers can objectively reflect the influence of the papers in academic exchanges, highly cited papers have recently become the standard for evaluating the scientific research level internationally [28,29,30].

Table 1 lists the top 10 highly cited papers, with citation frequencies of no less than 13. Moreover, the journal distribution of these papers is as follows: three papers, at the most, were acquired from Medicine and Philosophy, with ‘The new changes of physician–patient relationship in the network environment and its improvement measures’ being the most cited, with a citation frequency of 26. These papers classify the changes of professional–patient relations in the Internet era in China and propose measures on how to improve them.

These papers have significant research topics that have an essential impact and have played a significant role in promoting the development of the frontiers of the research topic. In addition, the research content of these papers is at the forefront in the field [29].

#### 3.1.4. Journal Distribution Analysis

Articles on professional–patient relations in the Internet era in China were published in 275 journals from 1998 to July 14, 2018, and all were written in Chinese. We listed these journals in the order of decreasing number of publication and then divided them into a nucleus of journals, with two groups containing approximately the same number of publications as the nucleus. Notably, *Medicine and Philosophy* is the most productive journal (shown in Table 2), which had published 15 articles about professional–patient relations in the Internet era in China. Combined with the highly cited paper analysis, we can infer that *Medicine and Philosophy* is one of the most significant core carriers of research on the topic.

As shown in Table 2, the nucleus, with 162 articles accounting for 31.06% of all articles, covers the top 22, or 8%, of the journals. The relationship between the number of journals in the nucleus and the two succeeding zones is approximately 1:3:32, which is in accordance with Bradford’s law of scattering [31]. In addition, most of the journals are about medicine and health technology, except for *Office Operations* and *Today’s Mass Media*.

#### 3.1.5. Research Institution Distribution Analysis

Scholars from 466 institutions have contributed to the research in China. The top ten institutions are the most productive, accounting for 11.78% of the total articles. As shown in Table 3, the Second Military Medical University performed well and was the most productive institution in the field, followed by the Huazhong University of Science and Technology and Mudanjiang Medical University. The other institutions were affiliated with different universities, except for Hunan Children’s Hospital. Similar to other research fields, universities are significant research forces.

### 3.2. High-Frequency Keyword Analysis

Keywords of an article provide information about its core content and also help in understanding the development of research topics over time [32]. Therefore, high-frequency keywords can reflect evolving research frontiers relating to some knowledge domains.

According to L. Egghe, the g-index focuses on the contribution of high-frequency keywords to all words, and can determine the sub-high frequency keywords and eliminate the role of low-frequency keywords [18]. Therefore, according to the definition of the g-index, the value of g is 22. Table 4 shows this result.

Table 5 lists the high-frequency keywords. The most frequent keyword is “professional–patient relations,” which is consistent with our theme. Keywords relating to professional–patient relations, such as “professional–patient communication” and “professional–patient dispute,” also appear in Table 5. These keywords have accounted for 37.89% of the total keywords, which has shown some research hotspots of the field to a certain extent.

### 3.3. Keyword Frequency Distribution Analysis

**Figure 6:**
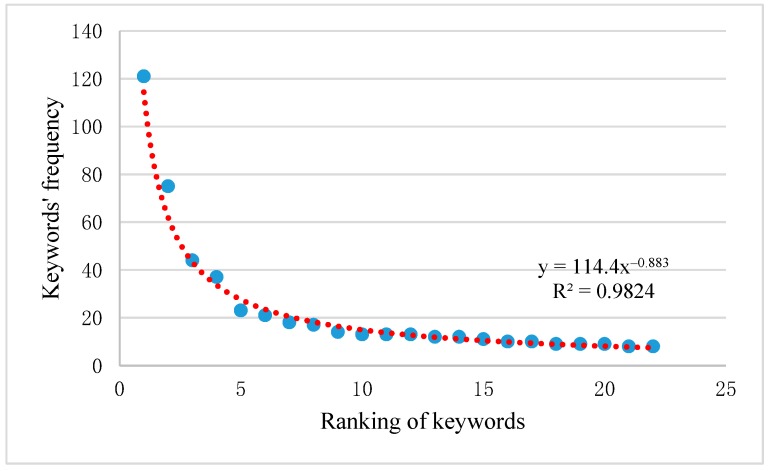
Distribution of the frequency of the keywords.

We collected a total of 1338 keywords in all 522 articles (2.56 keywords per article). Most of the keywords were used only once in the articles, whereas just a small number of keywords was used frequently. Furthermore, Figure 6 shows the distribution of the frequency of high-frequency keywords, with *R*^2^ = 0.9824. Therefore, we can infer that this approximately follows Zipf’s Law [33] with an exponent of −0.883, indicating that the research structure in this field is unevenly distributed [22].

### 3.4. Social Network Analysis

**Figure 7:**
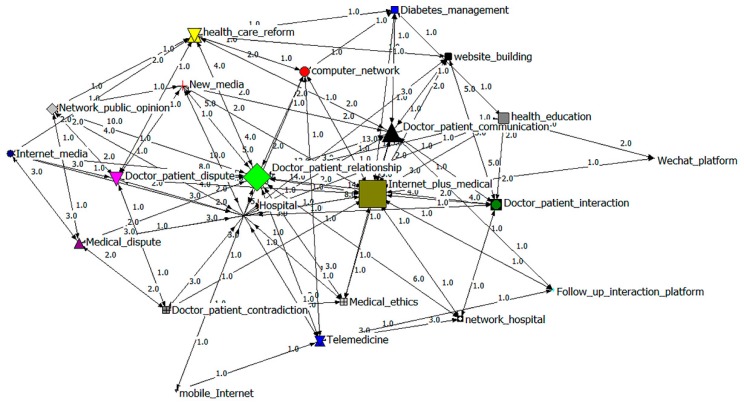
Social network maps of the original 22 × 22 co-occurrence matrix.

In accordance with the 22 × 22 co-occurrence matrix, a network map was drawn using Netdraw2.0 embedded in Ucinet6.0 (shown in Figure 7), reflecting the relationships between high-frequency keywords. The relative size of the nodes is proportional to the frequencies of the keywords, and the relative thickness of the lines is proportional to the correlations between keywords [21], which means that the thicker the line between two nodes, the closer the relationship between them [34]. For example, “professional–patient relations”, “Internet + medical”, “professional–patient communication”, and “hospital” have bigger nodes, indicating that they have higher frequencies. Moreover, the thick lines between two nodes, such as “professional–patient relations”, “hospital”, “professional–patient interaction”, and “website building”, imply their strong connections.

### 3.5. Hierarchical Cluster Analysis

**Figure 8:**
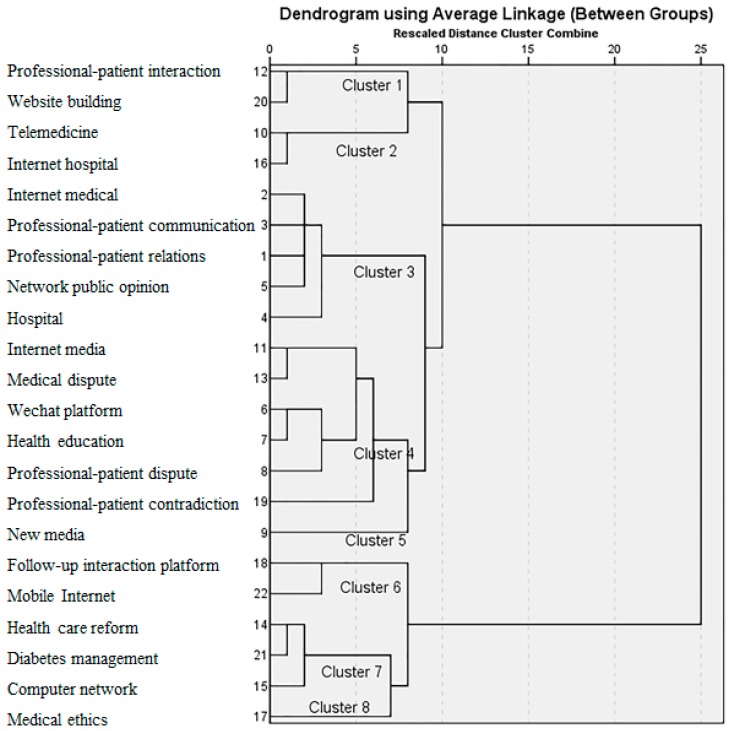
Eight clusters of 22 keywords.

Figure 8 shows the dendrogram of the hierarchical cluster analysis. We divided the 22 keywords into eight clusters, indicating that the research directions are broad and varied. The number on the vertical axis is the ranking number of the high-frequency keywords, whereas the numbers on the horizontal axis represent distances between two keywords. That is, the shorter the distance where two keywords come together, the closer their relationship. For example, the relationship between keywords 12 and 20 (professional–patient interaction and website building) is the closest of all the keywords. In addition, they combine into Cluster 1. Cluster 1 focuses on website building, especially for professional–patient interaction. Cluster 2, consisting of keywords 10 and 16, focuses on telemedicine, such as Internet hospital, which first appeared in China and subsequently all over the world [35]. Cluster 3 focuses on professional–patient communication and network public opinion. Cluster 4 focuses on professional–patient contradiction and health education in Internet media and social software platforms, such as the WeChat platform, including keywords 6, 7, 8, 11, 13, and 19. Cluster 5 focuses on new media. Cluster 6 focuses on a follow-up interaction platform with the development of mobile Internet. Cluster 7 focuses on healthcare reform, diabetes management, and computer network. Cluster 8 is related to medical ethics.

### 3.6. Strategy Diagram Analysis

We calculated the density and centrality of each cluster based on the 22 × 22 co-occurrence matrix [36] with Excel 2016 (shown in Table 6). Subsequently, we drew a strategic diagram with the origin (0.0064, 0.0893) (Figure 8). The strategic diagram clearly shows the research hotspots and trends by dividing these clusters into four quadrants.

**Figure 9:**
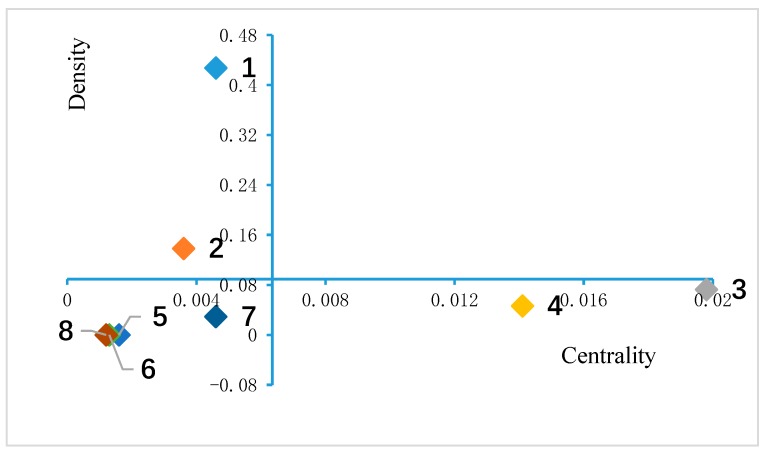
Strategic diagram.

Figure 9 shows that quadrant I has no cluster. The density and centrality of clusters in quadrant I are high, indicating that they are tightly connected, internally and externally, and thus can be regarded as the central topics of the field and tend to be mature. Therefore, the central topic of professional–patient relations in the Internet era in China has not appeared.

Clusters 1 and 2 are located in quadrant II with a low degree centrality but high density, suggesting that the cluster is actively developed internally but is rather peripheral to the network.

Clusters in quadrant III are clusters 5, 6, 7, and 8. Low density and centrality reflect that these clusters are weakly developed internally and are peripheral to the research network in China, often indicating that these topics are at the boundary of the field.

Clusters 3 (professional–patient communication and network public opinion) and 4 (professional–patient contradiction and health education in Internet media and social software platform) are located in quadrant IV, which has a high degree of centrality and low density. This cluster is the research core of the field in China but is weakly developed internally. In other words, the cluster may correspond to a newly appearing research theme.

## 4. Discussion

### 4.1. Key Findings

In this study, the methods mainly covered statistical, social network, hierarchical cluster, and strategic diagram analyses. On the basis of the results, we drew the following valuable conclusions.

First, the number of articles has risen continually since 1998, which follows the growth law of literature. Furthermore, the journal distribution follows Bradford’s law of scattering, and there are 22 journals in the core zone. As for classic literature, ‘The new changes of physician-patient relationship in the network environment and its improvement measures’, published by *Medicine and Philosophy* in 2013, classifies the new changes of the professional–patient relationship in the Internet era in China and proposes measures on how to improve it. The core author group has not been formed, but the author’s collaborative degree is on the rise. In addition, research institutions are distributed unevenly, and the Second Military Medical University remarkably topped all the institutions in its publication outputs during the past 21 years.

Second, we selected 22 keywords with high frequency (≥8) and the distribution of these keywords’ frequencies follows Zipf’s Law. These keywords, which are more active, better reflect the research hotspots and trends of relevant research in China to a great extent. Furthermore, we divided them into eight clusters, which focus on website building (especially for professional–patient interactions), telemedicine, professional–patient communication and network public opinion, professional–patient contradiction and health education, new media, follow-up interaction platforms, healthcare reform, computer networks, and medical ethics. Each cluster represents a research direction of professional–patient relations in the Internet era in China.

Finally, the major research topics have not yet formed and are in an imbalanced development on the whole. That is, quadrant I has no cluster, quadrants II and IV have two clusters, and quadrant III has four clusters. Specifically, topics in Cluster 3 (professional–patient communication and network public opinion using new media) and Cluster 4 (professional–patient contradiction and health education in Internet media and social software platforms) may be a newly appearing research theme with great potential for development.

We searched literature on PUBMED, without any result, using the following search strategy:

((((“Professional–Patient Relations” [Mesh]) and “Internet” [Mesh]) and “Bibliometrics” [Mesh]) not “China” [Mesh]).

There is no similar research using bibliometrics to study professional–patient relations in the Internet era in other geographical areas. Then, we obtained 157 review papers about professional–patient relations in the Internet era in other geographical areas with the following search strategy.

((((“Professional–Patient Relations” [Mesh]) and “Internet” [Mesh]) and “Review” [Publication Type]) not “China” [Mesh]).

After reading the abstracts of these review papers, we found that scholars in other geographical areas mainly focused on online health information seeking [37,38,39,40,41], social media use in healthcare [42], Internet-based interventions (IPIs) [43], online community [44,45], and e-health [46]. Compared to our results, scholars in other areas mainly focused on online health information seeking and Internet-based interventions. In addition, social media in healthcare, online community, and e-health obtained attention from scholars from both China and other geographical areas.

In summary, the present study provides the basis for a comprehensive understanding of professional–patient relations in the Internet era in China, which can be a potential guide for researchers in launching new projects in the future.

### 4.2. Limitations

Due to some constraints in the construction of any bibliometric map, future improvements are recommended to address the following limitations.

The limited scope of data collection in this study may have underrepresented publications in this domain, and some other document types, such as monographs, edited books, reports, and conference proceedings, may be valuable for analysis [47]. This exercise also demonstrated that relevant records can be missing if the query phrases for the topic search do not appear in subjects, titles, abstracts, and keywords [48]. However, we excluded non-Chinese papers, which may constitute a selection bias. The coverage of languages also causes intractable problems for bibliometrics [49].

We analyzed the degree of author collaboration. Other researchers can try collecting the locations of authors, draw their geographical distribution maps of cooperative networks, and examine the geographical distance between co-authors with the help of Google Earth. This information would be useful to describe the flow of knowledge and ideas through collaboration in the field.

## 5. Conclusions

Based on the hierarchical cluster analysis, the Internet hospital, as an emerging new form of telemedicine services, is developing at a substantial speed in China. It is an innovative practice of “Internet Plus Medical Health” with strong Chinese characteristics [50]. Furthermore, it is of great value for the improvement of professional–patient relations in China. With the Internet, people can overcome geographical obstacles and shatter time barriers to healthcare access. In big-city and top-flight hospitals, patients only get approximately two minutes to communicate with a doctor. However, visits to the Internet hospital last 10 min or more, and, as a result, patients’ satisfaction ratings are higher [51]. In addition, visits to the Internet hospital are cheaper than outpatient costs in a traditional hospital [35]. Therefore, the Internet hospital can help improve professional–patient relations significantly. This outpatient service is in use in Guangdong province, China, where the first officially approved “Internet hospital” went online on 25 October 2014 [35].

Nevertheless, the Internet hospital program is still in the exploratory stage, and quite a few problems remain to be solved, such as the incorporation of Internet medical services into health insurance programs, quality control, the applicability of Internet diagnoses for some diseases, possible medical disputes, and the long-term return on investment [35]. If the problems cannot be adequately solved, it may harm professional–patient relations in turn [52]. Therefore, the Internet hospital program needs to be further perfected in China.

According to the strategy diagram analysis, we discover two potential emerging research hotspots.

First, with the development of the Internet and information technology, increasingly more diverse ways for professional–patient communication are emerging. In addition, social media, especially WeChat and Microblog, has played an important role [53]. Based on the highly cited paper analysis, one paper on WeChat and Microblog exists. Moreover, the utilization of health social media can provide increased opportunities for communication, health promotion, and access to vital health information [54]. With social media platforms, we can improve professional–patient communication efficiency and relationships to a great extent. Therefore, the communication of doctors and patients through social media platforms may be a new trend for research in the future.

In recent years, incidents about the medical network public opinion have frequently appeared, causing irreversible loss and damage to society and people [55]. Furthermore, research about network public opinion has deepened, and findings have increased day by day. For example, among the top 10 highly cited papers (shown in Table 1), there are two papers about the network public opinion. Scholars have analyzed the evolution of network public opinion from different angles [56]. Compared with traditional news media, the dissemination of information on the network can be more timely and rapid [57,58]. Therefore, for medical institutes to maintain their image and improve professional–patient relations, learning how to predict potential negative network public opinion trends and deal with them timely and adequately is useful. It is necessary to establish early warning mechanisms and study the evolution laws of involved medical network public opinion events [59,60]. Medical institutes should formulate contingency plans to deal with potential network public opinion events. In addition, active cooperation with new media is helpful [59]. Above all, medical institutes must improve their quality of medical services [60] to fundamentally reduce adverse network public opinion events. The accurate prediction of and proper response to network public opinion trends has great significance in building harmonious professional–patient relations.

The shortage of medical resources, which is impossible to solve worldwide in the short term, causes the professional–patient contradiction. Therefore, it is fundamental to further promote the Health Care Reform and solve the problem of difficult and expensive access to medical services. With the implementation of China’s health care reform policy, patients’ feelings and medical treatment environment have been greatly improved. In addition, it is helpful for the improvement of professional–patient relations [61]. China’s health care reform will affect not only China’s future, but also the global healthcare model [62]. Improving health insurance coverage, reducing costs, and dealing with the huge challenge of disease is a problem facing the whole world. China’s health care reform will ultimately enrich the achievements of the global health care reform, which will be especially valuable for areas with a shortage of health resources.

Follow-up is an observational method used by hospitals for the patients who have visited the hospital, in which they regularly supervise the changes in patients’ conditions and guide their recovery by means of communication. It is helpful for the improvement of professional–patient relationships to build the follow-up interaction platform. It not only helps hospitals to supervise patients’ conditions and provide guidance, but also to improve the interaction between medical staff and patients. Moreover, it can be used to aid clinical research. However, according to the above results, we find that researchers pay more attention to the construction of platforms, while few explore how to ensure the effective management and use of platforms. Therefore, this may be a potential research theme [63].

## Figures and Tables

**Table 1:** Top 10 highly cited papers, with a citation frequency of no less than 13.

Papers	Journal	Citation Frequency
The new changes of physician-patient relationship in the network environment and its improvement measures	*Medicine and Philosophy*	26
The research and application on new pattern hospital customer relationship management platform under digital condition	*Chinese Hospitals*	18
Application of Internet phone intervention in health education for patients with hypertension	*Journal of Nursing*	17
Establishment and effect of diabetes management model based on network information community-hospital integration	*Chinese Community Doctors*	17
The negative impacts of network public opinion on the doctor-patient relationship and the countermeasures	*Medicine and Philosophy*	15
Legal problem brought by network medical treatment	*Medicine and Philosophy*	15
Application of WeChat in nursing work	*Digital Technology & Application*	14
Organization and management of building up digital hospital	*Hospital Administration Journal of Chinese People* *’s Liberation Army*	13
Application of microblog in medical practice	*Clinical Medicine & Engineering*	13
Network-based analysis of public sentiments on violent events in medical staff	*Chin J Med Libr Inf Sci*	13

**Table 2:** Top 22 journals on the topic.

Top Journals	Field	Publication
*Medicine and Philosophy*	Medicine and health technology	15
*Chinese Medical Ethics*	Medicine and health technology	13
*Chinese Hospitals*	Medicine and health technology	12
*Medical Information*	Medicine and health technology	11
*Chinese Journal of Hospital Administration*	Medicine and health technology	11
*Journal of Frontiers of Medicine*	Medicine and health technology	10
*Modern Hospital*	Medicine and health technology	7
*Medicine and Society*	Medicine and health technology	7
*Hospital Management Forum*	Medicine and health technology	7
*Chinese Journal of Health Informatics and Management*	Medicine and health technology	7
*Hospital Administration Journal of Chinese People* *’s Liberation Army*	Medicine and health technology	6
*China Hospital CEO*	Medicine and health technology	6
*Office Operations*	Economics and management science	5
*Jilin Medical Information*	Medicine and health technology	5
*Today* *’s Mass Media*	Information technology	5
*Journal of Modern Medicine & Health*	Medicine and health technology	5
*Health Care Guide*	Medicine and health technology	5
*China Digital Medicine*	Medicine and health technology	5
*China Health Industry*	Medicine and health technology	5
*Chinese Health Service Management*	Medicine and health technology	5
*China Medical Herald*	Medicine and health technology	5
*Chinese Hospital Management*	Medicine and health technology	5

**Table 3:** Top ten institutions.

Ranking	Institutions	Frequency	%
1	The Second Military Medical University	22	1.8003
2	Huazhong University of Science and Technology	17	1.3912
3	Mudanjiang Medical University	16	1.3093
4	Beijing University of Chinese Medicine	15	1.2275
5	Nanjing General Hospital of Nanjing Military Command, PLA	13	1.0638
6	Nanjing Medical University	13	1.0638
7	ZHONGSHAN Hospital	13	1.0638
8	Affiliated Hospital of Guilin Medical University	12	0.9820
9	XINQIAO Hospital, Third Military Medical University	12	0.9820
10	Hunan Children’s Hospital	11	0.9002

**Table 4:** Frequencies of keywords and their *g*-index (partly).

(Ranking)g	g^2^	Keywords	Frequency	Cumulative Frequency
1	1	professional–patient relations	121	121
2	4	Internet + medical	75	196
3	9	professional–patient communication	44	240
4	16	Hospital	37	277
……	……	……	……	……
21	441	Diabetes management	8	499
22	484	Mobile-Internet	8	507
23	529	Public hospital	8	515
24	576	Medicine	8	523
25	625	Health management	8	531
……	……	……	……	……

**Table 5:** High-frequency keywords and their frequencies (*g*-index = 22).

Ranking	Keywords	Frequency	%
1	professional–patient relations	121	9.04
2	Internet + medical	75	5.61
3	professional–patient communication	44	3.29
4	Hospital	37	2.77
5	Network public opinion	23	1.72
6	WeChat platform	21	1.57
7	Health education	18	1.35
8	professional–patient dispute	17	1.27
9	New media	14	1.05
10	Telemedicine	13	0.97
11	Internet media	13	0.97
12	professional–patient interaction	13	0.97
13	Medical dispute	12	0.90
14	Health care reform	12	0.90
15	Computer network	11	0.82
16	Internet hospital	10	0.75
17	Medical ethics	10	0.75
18	Follow-up interaction platform	9	0.67
19	professional–patient contradiction	9	0.67
20	Website building	9	0.67
21	Diabetes management	8	0.60
22	Mobile Internet	8	0.60

**Table 6:** Density and centrality of each cluster.

Cluster	Centrality(x)	Density(y)
1	0.0046	0.4274
2	0.0036	0.1384
3	0.0198	0.0725
4	0.0141	0.0465
5	0.0016	0
6	0.0013	0
7	0.0046	0.0294
8	0.0012	0
